# GLA Gene Mutation in Hypertrophic Cardiomyopathy with a New Variant Description: Is it Fabry's Disease?

**DOI:** 10.5935/abc.20190112

**Published:** 2019-07

**Authors:** Ândrea Virgínia Chaves-Markman, Manuel Markman, Eveline Barros Calado, Ricardo Flores Pires, Marcelo Antônio Oliveira Santos-Veloso, Catarina Maria Fonseca Pereira, Andréa Bezerra de Melo da Silveira Lordsleem, Sandro Gonçalves de Lima, Brivaldo Markman Filho, Dinaldo Cavalcanti de Oliveira

**Affiliations:** 1 Universidade Federal de Pernambuco - Hospital das Clínicas - Área Acadêmica de Medicina Clínica - Centro de Medicina Clínica - CCM, Recife, PE - Brazil; 2 Hospital Agamenon Magalhães, Recife, PE - Brazil; 3 Universidade Federal de Pernambuco - Grupo de pesquisa em Epidemiologia e Cardiologia (EPICARDIO), Recife, PE - Brazil; 4 Pós Graduação em Inovação Terapêutica (PPGIT) - Universidade Federal de Pernambuco, Recife, PE - Brasil; 5 Centro Universitário Mauricio de Nassau - Curso de Medicina, Recife, PE - Brasil; 6 Clínica de Doenças Metabólicas, Porto Alegre, RS - Brasil; 7 Cetogene AG, Rostock - Germany

**Keywords:** Fabry Disease/genetic, Cardiomyopathy, Hypertrophic, Hypertrophy, Left Ventricular, Glycosphingolipids

## Abstract

**Background:**

Fabry disease (FD) is an X-linked lysosomal storage disorder caused by mutations in the alpha galactosidase A gene (GLA) that lead to the enzymatic deficiency of alpha galactosidase (α-Gal A), resulting in the accumulation of globotriaosylceramide (Gb3) and globotriaosylsphingosine (lyso-Gb3), causing multiple organ dysfunctions.

**Objective:**

To perform GLA gene screening in a group of patients with echocardiographic diagnosis of hypertrophic cardiomyopathy (HCM).

**Methods:**

a cross-sectional study was conducted with HCM patients from a university hospital. Patients with coronary artery disease and valvulopathies were excluded. Mutation analysis of the GLA gene was performed. In male subjects, the analysis was performed after evidence of low α-Gal A activity.

**Results:**

60 patients with echocardiographic diagnosis of HCM were included. Age ranged from 12 to 85 years and 60% were women. Mean myocardial fibrosis percentage on MRI was 10.7 ± 13.1% and mean ventricular thickness was18.7 ± 6.7 mm. Four patients had the following GLA gene mutations: c.967C>A (p.Pro323Thr), not yet described in the literature; c.937G>T (p.Asp313Tyr); and c.352C>T (p.Arg118Cys). All patients had normal levels of lyso-Gb3 and non-ischemic myocardial fibrosis on magnetic resonance imaging; one patient had proteinuria and one patient had ventricular tachycardia.

**Conclusion:**

in this study, the frequency of mutation in the GLA gene in patients with HCM was 6.7%. A novel mutation in exon 6 of the GLA gene, c.967C>A (p.Pro323Thr), was identified. Patients with HCM may have GLA mutations and FD should be ruled out. Plasma (lyso-Gb3) levels do not seem to be sufficient to attain a diagnosis and organ biopsy should be considered.

## Introduction

Hypertrophic cardiomyopathy (HCM) is the most prevalent cardiopathy of genetic origin, caused by >1400 mutations in genes encoding proteins that are components of cardiac sarcomeres and other proteins of related structures, such as Z-discs and intercalated discs.^1-3^ Due to advances in molecular biology techniques, the differential diagnosis of HCM has been extended, and other genetic disorders that present with ventricular hypertrophy have been reported.^4^

Fabry disease (FD) is a rare X-linked genetic condition. It is caused by inborn errors in glycosphingolipid metabolism due to mutations in the gene encoding the enzyme α-galactosidase A (α-Gal A). Total or partial deficiency of this enzyme results in progressive accumulation of globotriaosylceramide (Gb3) and globotriaosylsphingosine (lyso-Gb3) in some tissues, particularly in the blood vessels, kidneys, and myocardium.^5-7^ More than 900 mutations with different effects on α-Gal A enzyme activity have been described.^8,9^

The incidence of FD is estimated at 1:40,000 to 117,000 individuals, and both male homozygous and female heterozygous may be affected.^5,6,8,9^ Two phenotypes are recognized: a classic form that is characterized by an early-onset with manifestations during childhood, and a late-onset form that frequently affects only one organ.^5,6,8^ Cardiac changes seem to be present in the early stages of life; however, it is usually not clinically detectable until the third or fourth decade of life. Deposits of glycosphingolipids in valves and coronary vessels are frequent, which may cause complete atrioventricular block, mitral insufficiency, left ventricular hypertrophy (LVH), or myocardial ischemia.^10^

Currently, specific mutations are associated with the cardiac variant showing myocardial hypertrophy that is clinically similar to HCM.^11,12^ Patients with FD are at risk for developing cerebrovascular disease (CVD), cardiac sudden death, and renal failure, and these patients can benefit from specific treatments.^13-15^

In this study, a screening for the *GLA* gene was performed in a group of patients with echocardiographic diagnosis of hypertrophic cardiomyopathy (HCM).

## Methods

### Subjects

A cross-sectional study was conducted in a convenience sample of patients with HCM treated at the cardiology outpatient clinic at a university hospital in Recife, Pernambuco, Brazil. All patients with an echocardiographic diagnosis of HCM based on the European Society of Cardiology^16^ criteria were included. Patients with coronary artery disease, valvulopathies and hypertensive cardiomyopathy were excluded.

### Definitions of HCM

A Transthoracic Echocardiography (TTE) was used to establish the diagnosis of HCM based on the following criteria: unexplained maximum ventricular thickness (MVT) ≥ 15 mm in any cardiac segment or a MVT ≥ 13 mm in a patient with family history of HCM. The obstructive presentation was defined by a left ventricular outflow gradient ≥ 30 mmHg at rest or after Valsalva maneuver or in orthostatism.^13,16^

### Echocardiographic assessment

The TTE was previously performed in all subjects to establish the diagnosis of HCM (Vivid 7 or Vivid E9 GE) with offline dataset analysis (EchoPac®, GE Healthcare, Little Chalfont, United Kingdom). The diameters of the interventricular septum and the left ventricular (LV) posterior wall, LV end diastolic volume, and LV end systolic volume were determined through M-mode or 2D imaging. The MVT of all segments were measured in the parasternal short axis. The ejection fraction (EF) and diastolic function were calculated using Simpson’s method and pulsatile Doppler, respectively.^17,18^

### Magnetic resonance imaging

Magnetic resonance imaging (MRI) was performed in all patients with *GLA* mutation to assess the presence of myocardial fibrosis using the delayed myocardial enhancement technique (DMET). A 1.5-T MRI scanner (MAGNETOM Essenza, Siemens Healthcare, Erlangen, Germany) with an eight-channel phased array coil was used. The images were acquired in the cardiac short axis, from the base to the apex, with 8-mm slices and 2-mm intervals using the T1-weighted echo-gradient sequence. An inversion recovery prepulse and adjusted inversion time were used to neutralize the myocardial signal. Image acquisition was started at approximately 8-10 min after the infusion of gadolinium contrast at a dosage of 0.2 mmol/kg. DMET increases the amount of contrast between the normal tissue (dark due to signal neutralization) and the fibrotic tissue (white due to enhancement of gadolinium in the T1-weighted sequence).^19^

### Electrocardiography and Dynamic electrocardiography (24-h Holter)

A resting 12-lead electrocardiogram was performed in all patients. A continuum digital recorder (*Cardio Light*®, Cardio Sistemas Comercial Industrial, São Paulo, Brazil) was used to record and analyze the Holter tests for 24 h. The electrodes were positioned in the electrocardiographic derivations MV1, MV4, and MV6. The results were analyzed using the *Cardiomanager* 540® (Cardio Sistemas Comercial Industrial, São Paulo, Brazil) software by an independent observer searching for cardiac arrhythmias.

### Molecular and enzymatic essays

All included patients underwent digit blood capillary puncture and blood samples were collected on filter paper. The samples were dried for 3-4h at room temperature, stored in a plastic envelop at 4°C, and sent to the Centogene Laboratory (Rostock, Germany).

Molecular analysis to determine *GLA* gene mutations was performed in the samples from the female subjects, whereas mutation analysis was performed after evidence of low α-Gal A activity in male subjects. The expression level and enzymatic activity of the biomarker lyso-Gb3 were identified through high-performance liquid chromatography and tandem mass spectrometry.

The *GLA* gene was analyzed using polymerase chain reaction (PCR) and sequencing of all coding regions and highly conserved exon-intron boundaries through next-generation sequencing with Illumina HiSeq (Illumina, California, USA). *GLA* gene analysis was performed in all patients with HCM.

### Statistical analyses

Data were analyzed descriptively using the Statistical Package for the Social Sciences version 20.0 (IBM Company, Armonk, NY, USA). Before analyzing the continuous variables, the data sets were tested for normality by performing the Shapiro-Wilk test. Normally-distributed continuous variables were presented with measures of central tendency and dispersion (mean and standard deviation), and categorical variables were described as absolute (n) and relative (%) frequencies. Comparative analysis was performed using Pearson’s chi-square test for categorical variables. Numerical variables were analyzed using the paired Student’s *t*-test. The significance level was defined as 5% throughout the entire statistical analysis (p < 0.05).

Paired student’s *t*-test was used to compare the baseline characteristics of both HCM and *GLA* mutation groups, and Pearson’s chi-square test was used to identify any associations between the clinical variables.

### Ethical standards

The study was approved by the Institutional Ethics Committee and was carried out according to Resolution 196/96 of the Brazilian National Health Council, which deals with the guidelines and standards for research involving human subjects. The investigation was also conducted in accordance with the Declaration of Helsinki. Informed consent was obtained from all patients before inclusion in this study.

## Results

We included 60 patients with an echocardiographic diagnosis of HCM that underwent molecular tests for FD. Their age ranged from 12 to 85 years (mean 42.3 ± 17), and 60% (n = 36) were women. Four patients, three of which were women, had *GLA* gene mutations, corresponding to 6.7% of our sample.

Syncope and dyspnea were the most frequent cardiac symptoms in all patients. Asymmetric septal hypertrophic cardiomyopathy was the most frequent type in patients without *GLA* mutations (61.5%) and in the *GLA* mutation group (50%). EF was similar in both groups. The most common electrocardiographic patterns of all patients were left ventricular hypertrophy (37.9%), atrioventricular block (13.8%) and left bundle branch block (10.3%). The clinical and epidemiological baseline characteristics of our sample are shown in Table 1.

**Table 1:** Clinical characteristics and complementary test results of patients

		HCM (n=56)	GLA mutation (n = 4)	p-value
Age (years)		42.3 ± 17.0	58.5 ± 15.2	0.11
Gender (female)		59.9%	75%	0.53
EF (%)†		67.6 ± 8.6	65.0 ± 4.2	0.43
Cardiac symptoms	Dyspnea	8%	25%	0.80
	Precordial pain	5%	-	
	Syncope	17.5%	50%	
	Palpitation	5%	-	
	Dizziness	7%	-	
Predominance of LVH †	Apical	5.56%	25%	0.38
	Concentric	25%	25%	
	AS	61.1%	50%	
	AM	0.05%	-	
	PMH	2.78%	-	
MVT (mm) †		19.1 ± 6.4	18.7 ± 0.9	0.98
Fibrosis on MRI (%)		11.0 ± 13.9	12.0 ± 11.6	0.82
ECG alteration	LVO	38.5%	100%	0.83
	LAE	10.3%	-	
	AVR	28.6%	-	
	LBBB	10.3%	25%	
	AVB	5.1%	50%	
	QRSFrag	20.5%	-	

HCM: hypertrophic cardiomyopathy; EF: ejection fraction; LVH: left ventricular hypertrophy; AS: Asymmetric septal; AM: anteromedial; PMH: papillary muscle hypertrophy; MVT: maximum ventricular thickness; MRI: magnet resonance imaging; ECG: electrocardiogram; LVO: left ventricular outflow tract; LAE: left atrium enlargement; AVR: abnormal ventricular repolarization; LBBB: left bundle branch block; AVB: atrioventricular block; QRSFrag: QRS fragmentation.

† measurement via transthoracic echocardiography (TTE).

**Figure 1:**
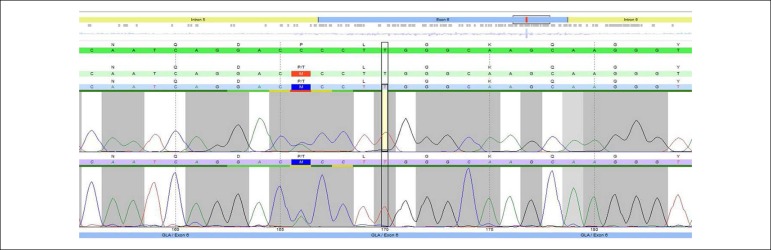
Chromatogram of the novel GLA gene mutation: c.967C>A (p.Pro323Thr).

Three mutations with heterozygote variants were found: c.967C>A (p.Pro323Thr), which is a novel mutation not yet described in literature (Figure 1); c.937G>T (p.Asp313Tyr); and c.352C>T (p.Arg118Cys). One homozygous variant was also found: c.352C>T (p.Arg118Cys). Five male patients underwent molecular analysis due to low α-Gal A enzyme activity; however, none of them had *GLA* gene mutations. The clinical and epidemiological characteristics of the patients with *GLA* gene mutations are described in Table 2.

**Table 2:** Clinical characteristics and complementary test results of FD patients

	Patient 1	Patient 2	Patient 3	Patient 4
Age (years)	69	39	72	53
Gender	F	F	F	M
Cardiac symptoms	Syncope	Syncope, dyspnea	No	No
Extracardiac symptoms	TIA	Acroparesthesia, intolerance to heat/cold, mood changes	Acroparesthesia, arteria thrombosis	ICVA
ECG	RHR, LVO, FDAVB, LBBB	RHR, LVO	RHR, LVO, FDAVB	RHR, LVO
24h Holter	8 episodes of VT, 677 PVC	No arrhythmias	Paroxysmal AFib	No arrhythmias
EF†	67%	60%	70%	65%
Predominance of LVH†	Apical	Asymmetrical septal hypertrophy	Asymmetrical septal hypertrophy	Concentric
MVT (mm)†	19	20	18	18
Diastolic disfunction†	Mild	Pseudonormal	Mild	Mild
LVOTO†	No	No	No	No
Fibrosis on MRI	6%	28%	13%	1.36%
FSD	Yes	Yes	No	No
ICD	Yes	Yes	No	No
GLA gene mutation	c.967C>A (p. Pro323Thr)	c.937G>T (p.Asp313Tyr)	c.352C>T (p.Arg118Cys)	c.352C>T (p.Arg118Cys)
High lyso-Gb3	No	No	No	No
Low α-Gal A	NM	NM	NM	Yes
Proteinuria	No	No	Yes	No

F: female; M: male; TIA: transitory ischemic attack; ICVA: ischemic cerebrovascular accident; ECG: electrocardiogram; RHR: regular heart rate; LVO: left ventricular overload; FDAVB: first degree atrio-ventricular block; LBBB: left bundle branch block; VT: ventricular tachycardia, PVC: premature ventricular contraction; AFib: atrial fibrillation; EF: ejection fraction; LVH: left ventricular hypertrophy; MVT: maximum ventricular thickness; LVOTO: left ventricular outflow tract obstruction; MRI: magnetic resonance imaging; FSD: family sudden death; ICD: implantable cardioverter defibrillator; NM: not measured.

† measurement via transthoracic echocardiography (TTE).

## Discussion

In our sample of 60 patients with HCM, we determined through molecular tests that the prevalence of *GLA* mutation was 6.7%. Additionally, we found a new mutation in the *GLA* gene. Enzyme replacement was started in one patient with *GLA* mutation.

FD has X-linked inheritance. The classic X-linked disorder generally shows a vertical transmission, in which heterozygous females transmit the allele down to their offspring. The majority of mutations in X-linked genes result in diseases that will only occur in males. However, some of the X-linked diseases show different rates of penetrance and expressivity in both genders. In FD, heterozygous individuals (females) are usually affected, but tend to have a milder and more variable phenotype than homozygous ones (males).^20^

The prevalence of FD is estimated to be 0.02-0.09:10,000 in the overall population, though molecular screening in newborns suggests a higher prevalence.^21,22^ Regarding the cardiac variant, the prevalence may be as high as 12%, depending on the method used.^23-26^

The mean maximum ventricular thickness (MVT) in patients with GLA mutation in our sample was greater than that in previous studies. The mean MVT was 11.6 ± 3.3 mm in the study by Koskenvuo et al.,^27^ and only eight patients had LVH. The mean MVT was 16.7 ± 1.9 mm in the study by Takenaka et al.,^28^ which is closer to that shown in this study. Patients with GLA mutation were screened from an HCM sample, which may explain the tendency for the higher mean values of MVT.

In our study, patients with GLA mutations had an older mean age. This finding could perhaps be attributed to the fact that the cardiac variant of FD has a later onset.

Regarding the hypertrophy pattern, FD is predominantly associated with concentric hypertrophy.^29^ However, some studies found a higher prevalence of the asymmetrical variety,^25^ which was in accordance with our results. This variety is usually associated with severe presentations of the disease.^25,29^

Atrioventricular block was present in the electrocardiographic (ECG) findings in two patients with *GLA* mutation (50%), which is in accordance with the findings from previous studies.^29^ However, repolarization abnormalities were common in patients with HCM, but not in patients with FD, as expected. These abnormalities are associated with mild concentric hypertrophy.^30^ In our study, only one patient showed concentric hypertrophy, which may explain the absence of repolarization abnormalities in the ECG results of our patients with *GLA* mutation.

The mean myocardial fibrosis percentage on MRI was 12±11.6% and 10.5±13.7% in patients with *GLA* mutation and no mutation, respectively. However, there was no statistically significant differences between the compared groups (p = 0.85). Although the clinical presentation of FD has been well described, the physiopathological mechanism linking the intracellular accumulation of Gb3 to tissue damage and clinical manifestations is still not well established.^31^ It is hypothesized that intracellular storage of Gb3 triggers important physiopathological cascades related to inflammatory processes, leading to myocardial cell changes and, over time, to fibrosis.^31-33^

*GLA* mutation was not associated with any specific late gadolinium enhancement (LGE) pattern on MRI. Patient 2 shows LGE in the basal midventricular septum, anterior septum, lateral inferior and apical segments of the left ventricle. Patient 3 shows LGE in the interventricular septum, inferior wall and in the right ventricle insertions. Patient 4 shows LGE in the basal segments of the inferior septum and inferior wall segments of the right ventricle. Information about LGE in patient 1 was not available.

One of the variants found has not been described in previous studies and in the *Exome Aggregation Consortium, Exome Sequencing Project* or 1000 *Genomes Browser* databases. The variant, c.967C>A (p.Pro323Thr), is located in exon 6 of the *GLA* gene. This mutation results in the substitution of a threonine amino acid by a proline amino acid at position 323 of the protein. This position shows a highly conserved nucleotide and a highly conserved amino acid, with moderated physico-chemical differences between the proline and threonine amino acids. It has characteristics of pathogenicity based on the analysis using *Polyphen-2, SIFT, MutationTaster,* and *Align-GVGD* software. Patient 1, the carrier of this mutation, had severe cardiac defects, manifested as arrhythmia and hypertrophy, with no other explainable causes.

The variant 352C>T (p.Arg118Cys) in exon 2 of the *GLA* gene was initially described to be pathogenic by Spada et al.^34^ According to Ferreira et al.,^35^ the moderated enzymatic deficiency related to p.Arg118Cys may not be sufficient to cause major complications of FD, suggesting low pathogenicity.^35^ This mutation was found in two unrelated patients (patients 3 and 4), both with LVH. Patient 3 also showed first-degree atrioventricular block and paroxysmal atrial fibrillation on ECG, as well as proteinuria. Patient 4 had a history of ischemic stroke. Despite the controversy over the pathogenicity of this variant, the authors believe it may cause specific organ manifestations, such as cardiac and cerebral involvement.

With regard to the third variant found in exon 6 of the GLA gene, GLA c.937G>T (p.Asp313Tyr) in patient 2, there are also contradictory results about its pathogenicity. Some studies showed that genotype D313Y is not responsible for severe organic lesions similar to those associated with the well-established genotypes of classic FD.^36^

Lenders et al.^36^ and Niemann et al.,^37^ reported that the presence of this variant is potentially associated with important white matter lesions in the central nervous system.^36,37^ The patient had severe cardiac hypertrophy, a history of uncontrolled systemic arterial hypertension, and complained of generalized pain with emotional lability. She has been receiving enzymatic replacement therapy for six months and has shown significant improvement of symptoms and blood pressure control.

The study of variants c.937G>T (p.Asp313Tyr) and c.352C>T (p.Arg118Cys) show contradictory results in the literature, but the authors believe they are pathogenic. The variant p. Asp313Tyr, found in this group, was identified in members of the same family and all follow the X-linked inheritance, with important cardiac hypertrophy and symptoms. Regarding the p.Arg118Cys, which has also shown controversial results in the literature, it was found in two patients from different families in this group. One is a homozygous male with cardiac hypertrophy and ischemic stroke; the other one is a heterozygous female patient also with cardiac hypertrophy, arterial thrombosis and proteinuria. The authors are currently working with the objective of gathering more evidence about the pathogenicity of these variants in the other family members. The new variant found, c.967C>A (p. Pro323Thr) seems to be pathogenic according to the analysis carried out with Polyphen-2, SIFT, MutationTaster and Align-GVGD software. As stated before, the patient is a heterozygous female with cardiac hypertrophy and transitory ischemic attack and investigation of the family members suggest pathogenicity.

Although the 352C> T (p.Arg118Cys) and c.937G> T (p.Asp313Tyr) variants are controversial as to pathogenicity, the families' heredogram confirms an X-linked inheritance. One of the most relevant results of the study was the identification of a new mutation in the *GLA* gene that seems to be pathogenic, in addition to the identification of 14 other carriers among the relatives of the four index patients. The probability of performing enzyme replacement therapy and pharmacological chaperones emphasizes the importance of an early diagnosis of FD^38-40^ and the search to identify the pathogenicity of the variants found.

### Limitations

The main limitations of our study were: the absence of sample calculation, the small sample size due to the disease rarity and the small number of patients with *GLA* mutations (statistical analyses were limited). Also, molecular analysis of sarcomeric genes in patients with HCM was not performed.

T1 mapping was performed on MRI images only for patient 4. At the time of the other scans, MRI T1 mapping was not available at our institution.

The authors understand that a renal or cardiac biopsy could be performed to confirm pathogenicity for those uncertain variants.

## Conclusion

In this study, the frequency of mutations in the *GLA* gene in patients with hypertrophic cardiomyopathy was 6.7%. A novel mutation in exon 6 of the *GLA* gene, c.967C>A (p.Pro323Thr), was identified. Patients with HCM may have *GLA* mutations and Fabry disease should be ruled out. Plasma lyso-GB3 levels do not seem to be sufficient to attain a diagnosis, and organ biopsy should be considered.

## References

[1] Baptista A, Magalhães P, Leão S, Carvalho S, Mateus P, Moreira I (2015). Screening for fabry disease in left ventricular hypertrophy:
documentation of a novel mutation. Arq Bras Cardiol.

[2] Boggio P, Luna PC, Abad ME, Larralde M (2009). Doença de Fabry. An Bras Dermatol.

[3] Niemann M, Weidemann F (2013). Echocardiography in Fabry disease. Cardiogenetics.

[4] Mattos BP, Torres MAR, Freitas VC (2008). Diagnostic evaluation of hipertrophic cardiomyopathy in its
clinical and preclinical phases. Arq Bras Cardiol.

[5] Albuquerque CV (2012). Anderson Fabry's disease: cardiac manifestations. Rev Bras Ecocardiogr Imagem Cardiovasc.

[6] Gómez MG, Varas C, Morales M, Bonacic F, Alvarez M, Rojas A (2013). Cardiac involvement in patients with Fabry's
disease. Rev Chil Cardiol.

[7] Kaminsky P, Noel E, Jaussaud R, Leguy-Seguin V, Hachulla E, Zenone T (2013). Multidimensional analysis of clinical symptoms in patients with
Fabry's disease. Int J Clin Pract.

[8] Hughes DA (2016). Fabry disease: will markers of early disease enable early
treatment and better outcomes?. Curr Opin Cardiol.

[9] Stenson PD, Mort M, Ball E V, Evans K, Hayden M, Heywood S (2017). The Human Gene Mutation Database: towards a comprehensive
repository of inherited mutation data for medical research, genetic
diagnosis and next-generation sequencing studies. Hum Genet.

[10] Linhart A, Elliott PM (2007). The heart in Anderson-Fabry disease and other lysosomal storage
disorders. Heart.

[11] Csányi B, Hategan L, Nagy V, Obál I, Varga ET, Borbás J (2017). Identification of a novel GLA gene mutation, p.Ile239Met, in
Fabry Disease with a predominant cardiac phenotype. Int Heart J.

[12] Hsu TR, Hung SC, Chang FP, Yu WC, Sung SH, Hsu CL (2016). Later onset Fabry disease, cardiac damage progress in
silence. J Am Coll Cardiol.

[13] Banikazemi M, Bultas J, Waldek S, Wilcox WR, Whitley CB, McDonald M (2007). Agalsidase-beta therapy for advanced Fabry disease: a randomized
trial. Ann Intern Med.

[14] Germain DP, Charrow J, Desnick RJ, Guffon N, Kempf J, Lachmann RH (2015). Ten-year outcome of enzyme replacement therapy with agalsidase
beta in patients with Fabry disease. J Med Genet.

[15] Wu JC, Ho CY, Skali H, Abichandani R, Wilcox WR, Banikazemi M (2010). Cardiovascular manifestations of Fabry disease: relationships
between left ventricular hypertrophy, disease severity, and
alpha-galactosidase A activity. Eur Heart J.

[16] Elliott PM, Anastasakis A, Borger MA, Borggrefe M, Cecchi F, Task Force members (2014). 2014 ESC Guidelines on diagnosis and management of hypertrophic
cardiomyopathy: the Task Force for the Diagnosis and Management of
Hypertrophic Cardiomyopathy of the European Society of Cardiology
(ESC). Eur Heart J.

[17] Nagueh SF, Smiseth OA, Appleton CP, Byrd 3rd BF, Dokainish H, Edvardsen T (2016). Recommendations for the evaluation of left ventricular diastolic
function by echocardiography: an update from the American Society of
Echocardiography and the European Association of Cardiovascular
Imaging. J Am Soc Echocardiogr.

[18] Steeds RP, Garbi M, Cardim N, Kasprzak JD, Sade E, Nihoyannopoulos P (2017). EACVI appropriateness criteria for the use of transthoracic
echocardiography in adults: a report of literature and current practice
review. Eur Heart J Cardiovasc Imaging.

[19] Sara L, Szarf G, Tachibana A, Shiozaki AA, Villa AV, Oliveira AC (2014). II Diretriz de Ressonância Magnética e Tomografia
Computadorizada Cardiovascular da Sociedade Brasileira de Cardiologia e do
Colégio Brasileiro de Radiologia. Arq Bras Cardiol.

[20] Pinto LL, Vieira TA, Giugliani R, Schwartz IV (2010). Expression of the disease on female carriers of X-linked
lysosomal disorders: a brief review. Orphanet J Rare Dis.

[21] Mechtler TP, Stary S, Metz TF, De Jesús VR, Greber-Platzer S, Pollak A (2012). Neonatal screening for lysosomal storage disorders: feasibility
and incidence from a nationwide study in Austria. Lancet.

[22] Hwu WL, Chien YH, Lee NC, Chiang SC, Dobrovolny R, Huang AC (2009). Newborn screening for Fabry disease in Taiwan reveals a high
incidence of the later-onset GLA mutation c.936+919G&gt;A
(IVS4+919G&gt;A). Hum Mutat.

[23] Hagege AA, Caudron E, Damy T, Roudaut R, Millaire A, Etchecopar-Chevreuil C (2011). Screening patients with hypertrophic cardiomyopathy for Fabry
disease using a filter-paper test: the FOCUS study. Heart.

[24] Monserrat L, Gimeno-Blanes JR, Marín F, Hermida-Prieto M, García-Honrubia A, Pérez I (2007). Prevalence of Fabry disease in a cohort of 508 unrelated patients
with hypertrophic cardiomyopathy. J Am Coll Cardiol.

[25] Chimenti C, Pieroni M, Morgante E, Antuzzi D, Russo A, Russo MA (2004). Prevalence of Fabry disease in female patients with late-onset
hypertrophic cardiomyopathy. Circulation.

[26] Sachdev B, Takenaka T, Teraguchi H, Tei C, Lee P, McKenna WJ (2002). Prevalence of Anderson-Fabry disease in male patients with late
onset hypertrophic cardiomyopathy. Circulation.

[27] Koskenvuo JW, Engblom E, Kantola IM, Hartiala JJ, Saraste A, Kiviniemi TO (2009). Echocardiography in Fabry disease: diagnostic value of
endocardial border binary appearance. Clin Physiol Funct Imaging.

[28] Takenaka T, Teraguchi H, Yoshida A, Taguchi S, Ninomiya K, Umekita Y (2008). Terminal stage cardiac findings in patients with cardiac Fabry
disease: An electrocardiographic, echocardiographic, and autopsy
study. J Cardiol.

[29] Yousef Z, Elliott PM, Cecchi F, Escoubet B, Linhart A, Monserrat L (2013). Left ventricular hypertrophy in Fabry disease: a practical
approach to diagnosis. Eur Heart J.

[30] Goldman ME, Cantor R, Schwartz MF, Baker M, Desnick RJ (1986). Echocardiographic abnormalities and disease severity in Fabry's
disease. J Am Coll Cardiol.

[31] De Francesco PN, Mucci JM, Ceci R, Fossati CA, Rozenfeld PA (2013). Fabry disease peripheral blood immune cells release inflammatory
cytokines: role of globotriaosylceramide. Mol Genet Metab.

[32] Seydelmann N, Wanner C, Störk S, Ertl G, Weidemann F (2015). Fabry disease and the heart. Best Pract Res Clin Endocrinol Metab.

[33] Biancini GB, Vanzin CS, Rodrigues DB, Deon M, Ribas GS, Barschak AG (2012). Globotriaosylceramide is correlated with oxidative stress and
inflammation in Fabry patients treated with enzyme replacement
therapy. Biochim Biophys Acta.

[34] Spada M, Pagliardini S, Yasuda M, Tukel T, Thiagarajan G, Sakuraba H (2006). High incidence of later-onset Fabry disease revealed by newborn
screening. Am J Hum Genet.

[35] Ferreira S, Ortiz A, Germain DP, Viana-Baptista M, Caldeira-Gomes A, Camprecios M (2015). The alpha-galactosidase A p.Arg118Cys variant does not cause a
Fabry disease phenotype: data from individual patients and family
studies. Mol Genet Metab.

[36] Lenders M, Duning T, Schelleckes M, Schmitz B, Stander S, Rolfs A (2013). Multifocal white matter lesions associated with the D313Y
mutation of the?-Galactosidase A gene. PLoS One.

[37] Niemann M, Rolfs A, Giese A, Mascher H, Breunig F, Ertl G (2013). Lyso-Gb3 indicates that the Alpha-Galactosidase A Mutation D313Y
is not clinically relevant for Fabry disease. JIMD Rep.

[38] Kampmann C, Perrin A, Beck M (2015). Effectiveness of agalsidase alfa enzyme replacement in Fabry
disease: cardiac outcomes after 10 years' treatment. Orphanet J Rare Dis.

[39] Biegstraaten M, Arngrímsson R, Barbey F, Boks L, Cecchi F, Deegan PB (2015). Recommendations for initiation and cessation of enzyme
replacement therapy in patients with Fabry disease: the European Fabry
Working Group consensus document. Orphanet J Rare Dis.

[40] Germain DP, Hughes DA, Nicholls K, Bichet DG, Giugliani R, Wilcox WR (2016). Treatment of Fabry's disease with the Pharmacologic Chaperone
Migalastat. N Engl J Med.

